# Lithobius (Ezembius) tetraspinus, a new species of centipede from northwest China (Lithobiomorpha, Lithobiidae)

**DOI:** 10.3897/zookeys.741.19980

**Published:** 2018-03-07

**Authors:** Sujian Pei, Yanmin Lu, Haipeng Liu, Xiaojie Hou, Huiqin Ma

**Affiliations:** 1 School of Life Sciences, Hengshui University, Hengshui, Hebei 053000, P. R. China; 2 Scientific Research Office, Hengshui University, Hengshui, Hebei 053000, P. R. China

**Keywords:** Chilopoda, Lithobius (Ezembius), NW China, Xinjiang Autonomous Region

## Abstract

Lithobius (Ezembius) tetraspinus **sp. n.** (Lithobiomorpha: Lithobiidae), recently discovered from Hami City, Xinjiang Autonomous Region, NW China, is described. Morphologically this species resembles L. (E.) sibiricus, Gersfeldt, 1858, but is distinguishable by a different coxal pore formula, absence of accessory spurs on leg 15, morphology of the second article of the female gonopod, and legs 14 plectrotaxy. A table of the main morphological characters of Chinese Lithobius (Ezembius) species is presented.

## Introduction

The centipede subgenus Lithobius (Ezembius) Chamberlin, 1919 accommodates a group of 58 species/subspecies mostly known from Asia, with little extension into north-western North America. Known species colonize a wide range of habitats, from arctic and sub-arctic to tropical and sub-tropical forests, to steppe and overgrazed stony areas of central Asia, to Himalayan montane forests, from sea shore up to 5500 m (Himalayas) (Zapparoli and Edgecombe 2011). Although the subgenus was formally proposed as new and described in 1923 (Chamberlin 1923), according to Jeekel (2005) its name had been already validated in 1919 (Chamberlin 1919). *Ezembius* is characterized by antennae with ca 20 articles; ocelli 1+4–1+20; forcipular coxosternal teeth usually 2+2; porodonts generally setiform, sometimes stout. Tergites are generally without posterior triangular projections; tarsal articulation of legs 1–13 is distinct. Female gonopods are with uni-, bi- or tridentate claw, and 2+2–3+3 (rarely 4+4) spurs (Zapparoli and Edgecombe 2011).

The myriapod fauna of China is still poorly known and very little attention has been paid to the study of Lithobiomorpha, with only 74 species/subspecies hitherto known from the country (Ma et al. 2014a, b, 2015; Minelli et al. 2016; Pei et al. 2014, 2015, 2016; Qin et al. 2014). Xinjiang Autonomous Region is among the poorly studied regions of China with only eight species at present registered from its territory (Ma et al. 2014b; Pei et al. 2015, 2016). Altogether, 18 species of Lithobius (Ezembius) have been recorded from China, only three of them from Xinjiang Autonomous Region. Here with a new species recently found in Balikun County is described.

## Materials and methods

All specimens were hand-collected under leaf litter or stones. The material was examined with the aid of a Motic-C microscope (Xiamen, China). The colour description is based on specimens in 75% ethanol, and the body length is measured from the anterior margin of the cephalic plate to the posterior margin of the postpedal tergite. Type specimens are preserved in 75% ethanol and deposited in the School of Life Sciences, Hengshui University, Hengshui, China (HUSLS). The terminology of the external anatomy follows Bonato et al. (2010). The following abbreviations are used throughout:

**T**, **TT** tergite, tergites;

**S, SS** sternite, sternites;

**C** coxa,

**Tr** trochanter,

**P** prefemur,

**F** femur,

**Ti** tibia,

**a** anterior,

**m** median,

**p** posterior.

## Taxonomic part

### Lithobiidae Newport, 1844

#### Lithobius (Ezembius) tetraspinus sp. n.

Taxon classificationAnimaliaLithobiomorphaLithobiidae

http://zoobank.org/846D108B-D41F-4C20-9161-DA2137A17977

Figs 1–7

##### Material examined.

**Figures 1–7.**
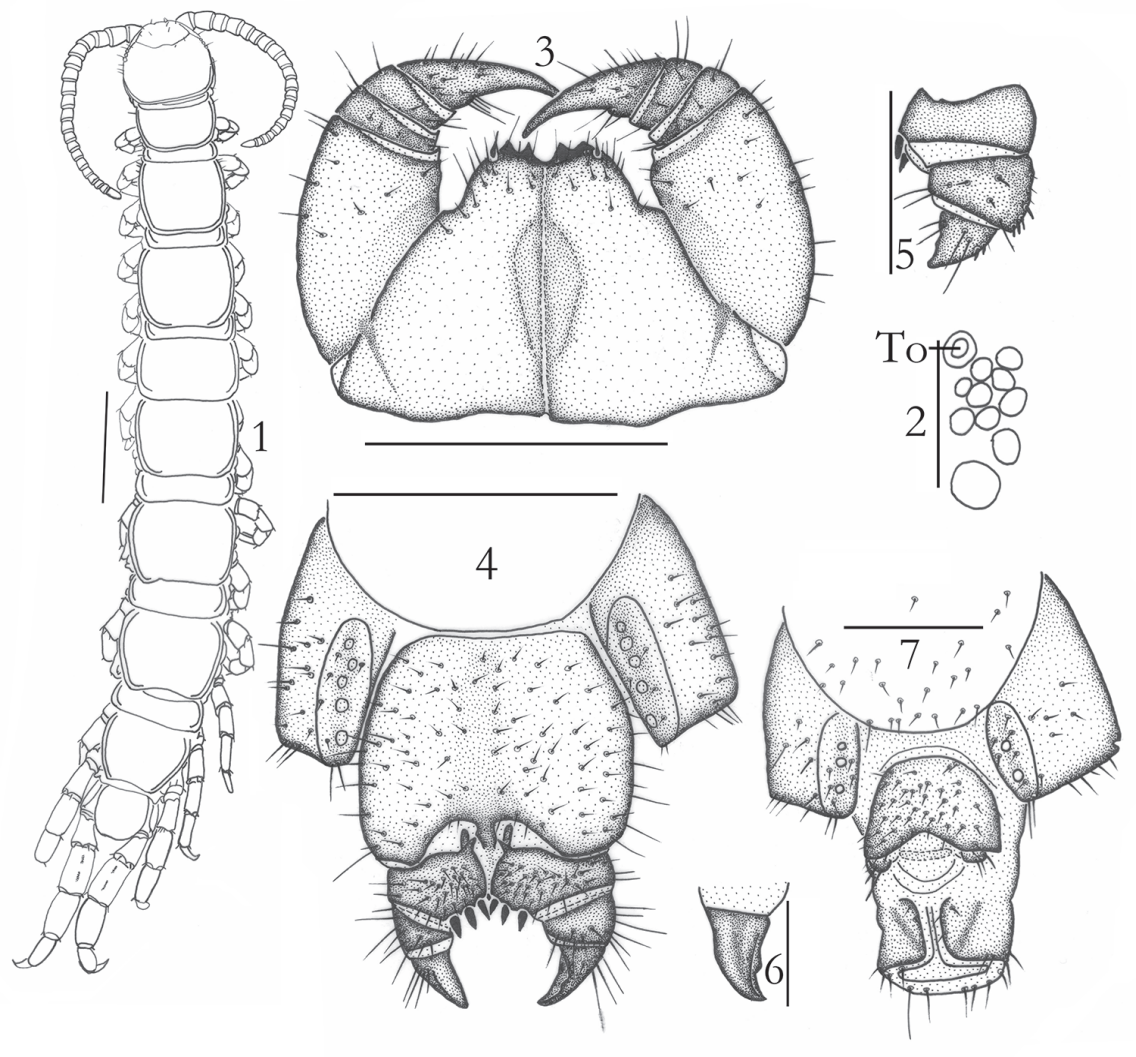
Lithobius (Ezembius) tetraspinus sp. n., 1–3 and 7 paratype, male: **1** habitus, dorsal view, scale bar 1 mm **2** ocelli and Tömösváry’s organ (To), lateral view, scale bar 250 μm **3** forcipular segment, ventral view, scale bar 500 μm; **4–6** holotype, female: posterior segments and gonopods, ventral view, scale bar 500 µm **5** posterior segments and gonopods, ventral view, scale bar 500 µm **6** posterior part of the external margin of second article of gonopods, ventral view, scale bar 250 μm **7** terminal claw of right gonopod, dorsal view, scale bar 250 µm.

**Holotype**: ♂ (Fig. 1), body length 11.7 mm, cephalic plate 1.10 mm long, 1.17 mm broad, Balikun County, Hami City, Xinjiang Autonomous Region, 43°06'N, 93°00'E, 968 m, a.s.l., 25 July 2006, leg. H. Ma, F. Zhang, S. Liu (HUSLS). **Paratypes**: 8 ♀♀, 1 ♂, same data as holotype (HUSLS).

##### Etymology.

The specific name refers to the second article of the female gonopods with four short, robust spines lying dorsally on the posterior part of the external margin.

##### Diagnosis.

A Lithobius (Ezembius) species with body length 9.6–13.3 mm, antennae composed of 19–22 articles, commonly 20+20; 8–10 ocelli on each side, arranged in 3 irregular rows, posterior two ocelli comparatively large; Tömösváry’s organ small, subequal in size to the adjoining ocelli; 2+2 coxosternal teeth; porodonts moderately thick, posterolateral to the lateralmost tooth; posterior angles of all tergites without triangular projections; coxal pores 2–5, oval to round; female gonopods commonly with 3+3 moderately large, coniform spurs; second article of female gonopods with four short, robust spines lying dorsally on the posterior part of the external margin; gonopods with a simple terminal article; male gonopods short and small, with 1–2 long setae on the terminal segment.

##### Description.

Body length 9.6–13.3 mm, cephalic plate 1.03–1.24 mm long, 1.06–1.31 mm wide.

*Colour*: basal antennal articles chocolate, distal articles gradually lighter, distalmost article yellow-brown. Tergites yellow-brown, TT 1 and 14 more darker. Cephalic plate and T 15 chocolate. Pleural region pale grey. Sternites pale yellow-brown. Distal part of forcipules red-brown, with basal and proximal parts of forcipules and forcipular coxosternite and sternite 15 yellow-brown. Legs 1–13 pale yellow-brown with greyish hue, legs 14 and 15 red-brown, tarsi of legs yellow-brown.

*Antennae*: 19–22 articles, commonly 20+20 (Fig. 1), only one specimen 20+25 articles; basal article longer than wide, second article markedly longer than wide, with following articles gradually shortening distally. Distalmost article 2.0–2.4 times as long as wide. Abundant setae on antennal surface, gradual increase in density of setae basally to distally to approx. 3–4^th^ article.

*Cephalic plate* smooth, convex, tiny setae emerging from pores scattered sparsely over the entire surface. Frontal marginal ridge of head with shallow anterior median furrow. Setae of various lengths scattered along the marginal ridge of the cephalic plate. Lateral marginal ridge discontinuous. Posterior margin continuous, straight (Fig. 1).

Eight to ten oval to rounded *ocelli* on each side (Fig. 2), arranged in three irregular rows; posterior two ocelli large; ocelli adjacent to the Tömösváry organ slightly small. Seriate ocelli domed, translucent, usually darkly pigmented.

*Tömösváry organ* at anterolateral margin of the cephalic plate, moderately smaller, subequal in size to the adjacent ocelli (Fig. 2-To).

*Coxosternite* subtrapezoidal (Fig. 3), anterior magin narrow, lateral margins of the coxosternite slightly longer than medial margins. Median diastema moderately deep, V-shaped; anterior margin with 2+2 subtriangular slightly acute teeth. Porodont thick and strong, just posterolateral and separated from the lateral tooth, hardly bulged at base (Fig. 3). Scattered short setae on the ventral side of coxosternite, longer setae near the dental margin, more longer setae near the porodont. Forcipules and forcipular coxosternite without obvious special modifications.

All *tergites* smooth, without wrinkles, dorsum slightly convex, tiny setae emerging from pores scattered sparsely over the entire surface, near the margin with few long setae; T 1 narrower posterolaterally than anterolaterally, generally trapezoidal, narrower than the cephalic plate and T 3, cephalic plate slightly wider than T 3. Lateral marginal ridges of all tergites continuous. Posterior marginal ridges of TT 1 and 3 slightly concave, continuous, posterior marginal ridges of TT 5, 8, 10, 12 and 14 shallow concave, discontinuous. Posterior angles of tergites generally rounded, without triangular projections. Miniscule setae scattered sparsely over the surface, more numerous setae on anterior and posterior angles of each tergite, with 2–4 long setae on anterior angles and 2–3 long setae on posterior angles of each tergite.

*Sternites* smooth, trapezoidal, posterior side narrower than anterior. Setae emerging from sparsely scattered pores on the surface, a pair of approximate symmetrically arranged long setae on both anterior part and posterior part of each sternite. The setae obviously increase in number on S 15, scattered evenly over the surface.

*Legs* robust, tarsal articulation ill-defined on legs 1–13, well defined on legs 14–15. All legs with fairly long curved claws. Legs 1–14 with anterior and posterior accessory spurs; anterior accessory spurs moderately long and slender, forming a moderately small angle with the claw, posterior accessory spurs slightly more robust, forming a comparatively large angle with the claw. Dense glandular pore on the surface of prefemur, femur, tibia, and tarsi of legs 14 and 15. Leg pair 15 lacking accessory spurs. Long setae sparsely scattered over the surface of prefemur, femur, tibia, and tarsi of legs 1–13; more setae on the tarsal surface, many thicker setae scattered evenly over the tarsal surface, setae arranged in one row on the ventral surface of tarsi of legs 1–13, with setae significantly reduced on legs 14 and 15, no thicker setae and setae arranged in one row on the ventral surface of tarsi present. Legs 14 and 15 slightly thicker than the anterior pairs in the female, tarsus 1 3.7–4.7 times as long as wide in legs 15. Legs 15 significantly thicker and stronger than the anterior pairs in the male, with a central longitudinal discontinuous shallow groove on the dorsal of femur, and a black vertical line at the bottom; tarsus 1 3.8–4.3 times as long as wide in legs 15. Leg plectrotaxy as in table 1.

*Coxal pores* 2–5, round or slightly oval, variable in sizes, arranged in a row; usually 4555, 4554, rarely 3454, 3455, 3343 in females and 2332, 2333 in males. Coxal pore field set in a relatively shallow groove, the coxal pore-field fringe with prominence. Prominence with short to moderately long setae sparsely scattered over the surface.

**Male.** S 15 posterior margin narrower than anterior, posteromedially slightly convex, sparsely covered with long setae, more than the anterior; sternite of genital segment obviously smaller than the female, usually well sclerotized; posterior margin deeply concave between the gonopods, without medial bulge. Long setae scattered on the ventral surface of the genital segment, fewer setae near S 15, fringed with longer setae along the posterior margin. Gonopods short, appearing as a small hemispherical bulge, with 1–2 long setae, apically slightly sclerotized (Fig. 7).

**Table 1.** Leg plectrotaxy of L. (E.) tetraspinus sp. n.

Legs	Ventral					Dorsal				
	C	Tr	P	F	Ti	C	Tr	P	F	Ti
1			p	am	m			p	ap	a
2			mp	amp	m			(a)p	ap	ap
3			mp	amp	am			(a)p	ap	ap
4-10			mp	amp	am			ap	ap	ap
11			mp	amp	am			amp	ap	ap
12			amp	amp	am	m		amp	p	ap
13			amp	amp	am	m		amp	p	p
14		m	amp	am	a	m		amp	p	p
15		m	amp	am	a	m		amp	p	

Letters in brackets indicate variable spines.

**Table 2-1.** The main morphological characters of the known Chinese species of subgenus Lithobius (Ezembius) Chamberlin, 1919.

Characters	*anabilineatus*	*anasulcifemoralis*	*bidens*	*bilineatus*	*chekianus*	*gantoensis*	*giganteus*	*insolitus*	*irregularis*
Sources	Ma et al., 2015	Ma et al., 2013	Takakuwa, 1939	Pei et al., 2014	Chamberlin & Wang, 1952	Takakuwa & Takashima, 1949	Eason, 1986	Eason, 1993	Takakuwa & Takashima, 1949
Distribution	China S (Guangxi)	China S (Guangxi)	China S (Taiwan)	China S (Guangxi)	China S (Zhengjiang and Taiwan)	China NW (Shanxi)	China N (Inner Mongolia Autonomous region)	China S (Hongkong)	China W (Shanxi)
Body length (mm)	11.9–12.1	10.1–12.3	15.0	9.0–9.1	16.0	9.0	15.0–50.0	10.0–11.5	12.0
Number of antennal articles	23+23 articles in female, unkown in male	19+19–24+24, commonly 20+20	20–21	two specimens with 20+21, one specimen with 20+23	20+20	20–23	20+20	18+18–19+19	20+20
Number, arrangement and shape of the ocelli	5 – 6, in 2 rows	6, in 3 rows	7	5–6, in 2 rows	5, in 3 rows	6	6–10, in 2–3 rows	6–8, in 2 rows	7, in 2 rows
Posterior ocellus	round, large	oval to round, large	comparatively large	oval to rounded	oval to round, comparatively large	oval to round, comparatively large	oval to round, comparatively large	oval to round, comparatively large	round, comparatively large
Seriate ocelli	subequal, all ocelli domed, translucent, usually darkly pigmented.	the one near ventral margin moderately small, others almost equal	not reported	subequal, all ocelli domed, translucent,usually darkly pigmented	not reported	comparatively large	not reported	not reported	subequal
Tömösváry’s organ	round, smaller than the adjoining ocelli	moderately large, rounded, slightlylarger than the adjoining ocelli	at most same size as one ocellus	slightly larger than the adjoining ocelli	not reported	subequal in size to the adjoining medium large ocelli	slightly smaller than the adjoining ocelli	slightly smaller than the adjoining ocelli	same size as largest ocellus
Number and arrangement of coxosternal teeth	2+2, subtriangular	2+2, moderately blunt	2+2	2+2, slightly triangular	2+2	2+2, approximately sharp small	2+2	2+2, approximately sharp small	2+2, small
Porodont	long, lying posterolateral to the most lateral teeth	slender, lying posterolateral to the lateral most tooth, their basal moderately bulged	moderately long	thick and long, lying posterolateral to the lateral most tooth	not reported	not reported	not reported	slender, lying posterolateral to the lateral tooth, their basal slightly bulged	long, their basal slightly bulged
Tergites	smooth, backside slightly hunched	smooth	not reported	smooth, slightly hunched behind	not reported	smooth, without wrinkles	smooth, with slightly wrinkles	T1 smooth, other with wrinkles	smooth
Number of coxal pores	3–5, female 4454, 3554; male 4443, 4453	3–6, usually 4663, 5654, 5553,5563 and 5565	5(6)555	usually females 4554, 5565; males 4553, 4454	6655 or 7665	3333	3333, 4554, 4555, 4565, 5565 or 5566	3–6, male 3443; female 4454, 4555, 5555, 5565	3–10, female 3–6 in 12^th^ leg, 4–6 in 13^th^ leg, 7–10 in 14^th^ and 15^th^ leg
Shape of coxal pores	round or slightly ovate	round or slightly ovate	round	ovate	not reported	round	round	round	round
Tarsus 1–tarsus 2 articulation on legs 1–13	not well–defined	not well–defined	well–defined	not well–defined	not reported	not reported	well–defined	not defined	well–defined
Male 14^th^ leg	obvious thicker and stronger than other legs	markedly thicker and stronger than 1–13 legs, more thicker and stronger than female	not reported	distinctly thick and strong	not reported	not reported	not reported	distinctly thick and strong	not reported
Male 15^th^ leg	obvious thicker and stronger than other legs	markedly thicker and stronger than 1–13 legs, more thicker and stronger than female	not reported	distinctly thick and strong	not reported	not reported	not reported	distinctly thick and strong, with dark zones on dorsal of tibia	not reported
Dorsal sulci on male 14^th^ legs	absent	absent	not reported	with two, shallow longitudinal sulci	not reported	not reported	not reported	absent	not reported
Dorsal sulci on male 15^th^ legs	two distinct, shallow, dorsal sulci on the femur and tibia	with a distinct, shallow, dorsalsulci on the tibia	not reported	with two, shallow longitudinal sulci	not reported	not reported	not reported	absent	not reported
DaC spine	on 14^th^–15^th^ legs	on 14^th^–15^th^ legs	absent	on 4^th^–15^th^ legs	on 14^th^–15^th^ legs	absent	on 12^th^–15^th^ legs (on 11^th^ and 12^th^ legs sometimes present)	absent	on 13^th^–15^th^ legs
14^th^ accessory spur	anterior accessory spur reduced in size, only half the length of the posterior accessory spur	absent	not reported	anterior accessory spur absent	present	present	present	not reported	not reported
15^th^ accessory spur	absent	absent	not reported	anterior accessory absent	present	present	absent	absent	not reported
Number and shape of spurs on female gonopods	2+2 moderately small, blunt, coniform spurs, inner spur slightly smaller than the outer	2+2 moderately blunt, with conical spurs, inner spur slightly smaller	3+3 or 4+4, sharp	2+2 moderately small, blunt, coniform spurs, inner spur slightly smaller than the outer one	not reported	1+1, conical spurs	2+2	3+3, coniform spurs	2+2 or 2+3, moderately small, blunt, coniform spurs
Dorsal side of the second article of female gonopods	with one spine lying dorsally on its external margin	no striking features	not reported	with three short, robust setae lying dorsally on its external margin	not reported	not reported	with eight spines in two irregular rows lying dorsally on its external margin	not reported	not reported
Apical claw of female gonopods (and lateral denticles)	simple, there a small subtriangular teeth in the inner	apical claw dimidiate	simple, there a small sharply teeth in the inner	apical claw bipartite, and its inner aspect broader	not reported	simple	simple	simple	simple and broad
Male gonopods	short and small bulge, with one to two long setae, apically slightly sclerotised	with a small bulge, without setae and apically less sclerotised	hemispherical, with two long setae	short and small bulge, having a long seta, apically slightly sclerotised	not reported	not reported	not reported	not reported	not reported

**Table 2-2.** Range and main morphological characters of the known Chinese species of subgenus Lithobius (Ezembius) Chamberlin, 1919.

Characters	*laevidentata*	*lineatus*	*mandschreiensis*	*multispinipes*	*parvicornis*	*rhysus*	*sulcipes*	*sulcifemoralis*	*zhui*	*tetraspinus*
Sources	Pei et al., 2015	Takakuwa, 1939	Takakuwa, 1940	Pei et al., 2016	Zapparoli 1991	Attems, 1934	Attems, 1927	Takakuwa & Takashima, 1949	Pei et al., 2011	This paper
Distribution	China NW (Xinjiang Uygur)	China S (Taiwan)	China (Taiwan, Sichuan, Jiangsu, Heilongjiang, Jilin, Liaoning)	China NW (Xinjiang Uygur)	China S (Taiwan)	China S (Fujian and Taiwan)	China S (Taiwan)	China W (Shanxi)	China NW (Xinjiang Uygur)	China NW (Xinjiang Uygur)
Body length (mm)	9.6–13.3	18.0	22.0–23.0	11.6–22.6	16.0	15.0	Not reported	12.0	8.1–15.0	9.6–13.3
Number of antennal articles	19+19–21+21 commonly 20+20	19+19–21+21	20–28	commonly 20+20, (three specimens with 20+21, one specimen with 20+26 of 134 specimens)	20+20, 21+21	20+20 in female, 20+21 in male	19–22	20+20	20–24, commonly 20	19–22, commonly 20
Number, arrangement and shape of the ocelli	8–10, in 3 rows	8–11, in 3 rows	9–13, in 3 rows	8, in 3 rows	3–4, in 1 or 2 rows	8, in 4 rows	7, in 2 rows	6	10–13, in 3–4 rows	8–10, in 3 rows
Posterior ocellus	posterior two ocelli bigger than the seriate ocelli	comparatively small	comparatively large	two ocelli large, oval to rounded	comparatively large	comparatively large	comparatively large	all ocelli same size	comparatively large	two ocelli comparatively large
Seriate ocelli	other seriate ocelli slightly larger than theocelli adjoining to the ventral	not reported	same size of wath	the two near ventral margin moderately small, others almost equal	not reported	not reported	not reported	same size of wath	dorsal ones moderately large, those near ventral margin of ocellar field moderately small, others of moderate size	the adjoining Tömösváry organ slightly small
Tömösváry’s organ	subequal in size to the adjoining ocelli	same size as the adjoining ocelli	larger than the adjoining ocelli	slightly smaller than the adjoining ocelli	not reported	not reported	not reported	same size as ocelli	slightly larger than the adjoining ocelli	subequal in size to the adjoining ocelli
Number and arrangement of coxosternal teeth	2+2, approximately blunt	2+2, comparatively large	2+2, small and sharp	3+3, slightly triangular	2+2	2+2	2+2	2+2, small and sharp	2+2 moderately small and pointed	2+2 subtriangular slightly acute
Porodont	thick and long, lying posterolateral to the most lateral teeth	long and strong	lying posterolateral to the lateral most tooth	thick and long, lying posterolateral to thelateral most tooth	lying posterolateral to the most lateral teeth	not obvious	not reported	slender and long	moderately thick in basal, moderately pointed, just posterolateral to the lateral tooth	Porodonts thick and strong, just posterolateral and separated from the lateral tooth,
Tergites	smooth, without wrinkles, backside slightly hunched	smooth	smooth, without wrinkles	smooth, without wrinkles and slightly hunched behind	smooth	With shallow wrinkles	Smooth, posterior angles slightly triangular in T14	not reported	smooth, without wrinkles, backside slightly hunched	smooth, without wrinkles, dorsum slightly convex
Number of coxal pores	2–5, female commonly 4555, 4554, sometime 3454, 3455, 3343. male commonly 2332, 2333, sometime 3444, 3333	6–7, usually 66(7)6	776(7)5(6)	3–5, 4555, 5555, 4444, 4455 (females) and 4444, 3344 (males)	3334	6554	4554	5555	2–4, 3444, 3344, 3443, 3333 in female, and 3443, 2343, 2433, 2333 in male.	usually 4555, 4554, rarely 3454, 3455, 3343 in females and usually 2332, 2333, rarely 3444, 3333 in males
Shape of coxal pores	round or slightly ovate	round to ovate	round or ovate	round to ovate	not reported	round	round	round	round or slightly ovate	round or slightly oval
Tarsus 1–tarsus 2 articulation on legs 1–13	not well–defined	well–defined	well–defined	well–defined	not reported	not reported	well–defined	well–defined	well–defined	ill–defined
Male 14^th^ leg	remarkably thicker and stronger than 1–13 legs,	not reported	not reported	thick and strong	not reported	not reported	not reported	thick and strong	moderately thicker and stronger	significantly thicker and stronger
Male 15^th^ leg	markedly thicker and stronger than in 1–13 legs	not reported	not reported	thick and strong	not reported	femur and tibia thicker	femur and tibia thicker	thick and strong	thicker and stronger, with a circular protuberance on distal end of tibia	significantly thicker and stronger
Dorsal sulci on male 14^th^ legs	absent	absent	not reported	absent	not reported	not reported	present on the femur	present on the femur and tibia	absent	absent
Dorsal sulci on male 15^th^ legs	with a distinct, shallow, dorsal sulci on the tibia	not reported	not reported	absent	not reported	not reported	present on the femur and tibia	present on the femur and tibia	absent	present on the femur
DaC spine	on 12^th^–15^th^ legs	on 14^th^–15^th^ legs	on 12^th^–15^th^ legs	on 11^th^–15^th^ legs, 9^th^–10^th^ sometimes present	not reported	on 15^th^ legs present	on 15^th^ legs present	absent	on 13^th^–15^th^ legs, 12^th^ sometimes present	on 12th–15th legs
14^th^ accessory spur	present	present	not reported	present	not reported	not reported	not reported	not reported	present	present
15^th^ accessory spur	anterior absent	present	not reported	absent	not reported	absent	not reported	not reported	absent	absent
Number and shape of spurs on female gonopods	3+4, or 4+4 small, blunt, coniform spurs, commonly with 3+3, inner spur smaller than the outer one	3+3 moderately sharp, slender conical spurs	3+3, same size	2+2, blunt, coniform spurs, with inner spur smaller than the outer one	2+2	2+2, slender	2+2, thick spurs	2+2, strong, long and sharp	2ﬂ 2 moderately long, coniform spurs, inner spur slightly smaller and more anterior than outer	3+3, few 3+4, only one 4+4 coniform spurs
dorsal side of the second article of female gonopods	with three long setae lying dorsally on its anterior external margin	not reported	not reported	with 3–4 long setae and 5–6 spines lying dorsally on its external margin	not reported	not reported	not reported	not reported	three spurs arranged in one irregular row on the dorsal terminal part	3 long setae and four short, robust spines lying dorsally on the posterior part of the external margin
Apical claw of female gonopods (and lateral denticles)	simple and broad	simple	simple	simple	simple	simple	dimidiate	simple	broad, and tridentate	simple, with a very small subtriangular blunt denticle on inner margin
Male gonopods	small bulge, with one to two long setae apically slightly sclerotised	hemispherical bulge,	without setae	hemispherical bulge, having a long seta, and apically slightly sclerotised	not reported	not reported	not reported	not reported	small bulge, with 1–2 long setae on surface, and terminal slightly sclerotised	small hemispherical bulge, with 1–2 long setae

**Female.** S 15 anterior margin broader than posterior, generally trapezoidal, posteromedially slightly convex. Short to long setae sparsely scattered on S 15 surface. Surface of the lateral sternal margin of genital segment well chitinized, posterior margin of genital sternite deeply concave between condyles of gonopods, except for a small, median linguliform bulge. Relatively long setae scattered over ventral surface of the genital segment, few setae near S 15. Gonopods: first article fairly broad, bearing 23–30 short to moderately long setae, arranged in four irregular rows; with 3+3, few 3+4, only one 4+4 moderately long and slender, coniform spurs, inner spur slightly smaller than the outer (Fig. 4); second article with 8–12 long setae, arranged in three irregular rows, with three long setae and four short, robust spines lying dorsally on the posterior part of the external margin; third article with 4–6 long posteroventral setae, and two short, robust spines lying dorsally on the posterior part of the external margin (Fig. 5). Third article of female gonopods with a simple apical claw with a very small subtriangular blunt denticle on the inner margin (Fig. 6).

##### Remarks.

The new species with 2+2 coxosternal teeth, 9–10 ocelli on each side of head, female gonopods with 3–4 moderately large, coniform spurs, and leg pair 15 lacking accessory spurs, is morphologically similar to Lithobius (Ezembius) sibiricus Gerstfeldt, 1858 from Mongolia and Russia, but is readily distinguished by having coxal pores arranged in a 2–5-formula in contrast to L. (E.) sibiricus with a coxal pore formula 5–8; the second article of the female gonopods with four short, robust spines lying dorsally on the posterior part of the external margin versus with eight short, robust spines lying dorsally on the posterior part of the external margin; lacking accessory spurs on legs 15^th^ versus having small accessory spurs on legs 15^th^; moreover, leg 14 plectrotaxy is distinctly different, 10311 (dorsal) and 01321 (ventral) compared to 10311 (dorsal) and 01332 (ventral).

##### Habitat.

The specimens were collected in a *Larix* forest at 950–1000 m alt. It inhabits moderately moist habitats under roadside stones and litter of the forest floor.

To assist in the identification of the Chinese species of Lithobius (Ezembius), the range and main morphological characters of the known species of the subgenus in the area is presented (Table 2). These characters are specific only to adults of the taxa occurring in China.

## Supplementary Material

XML Treatment for Lithobius (Ezembius) tetraspinus
